# A novel location classification system for Crohn’s disease based on small bowel involvement: a better predictor of disease progression

**DOI:** 10.1093/gastro/goae003

**Published:** 2024-02-11

**Authors:** Huili Guo, Jian Tang, Xiusen Qin, Minzhi Lin, Miao Li, Qingfan Yang, Zicheng Huang, Xiang Gao, Kang Chao

**Affiliations:** Department of Gastroenterology, The Sixth Affiliated Hospital, Sun Yat-sen University, Guangzhou, Guangdong, P. R. China; Guangdong Provincial Key Laboratory of Colorectal and Pelvic Floor Diseases, The Sixth Affiliated Hospital, Sun Yat-sen University, Guangzhou, Guangdong, P. R. China; Department of Gastroenterology, The Sixth Affiliated Hospital, Sun Yat-sen University, Guangzhou, Guangdong, P. R. China; Guangdong Provincial Key Laboratory of Colorectal and Pelvic Floor Diseases, The Sixth Affiliated Hospital, Sun Yat-sen University, Guangzhou, Guangdong, P. R. China; Guangdong Provincial Key Laboratory of Colorectal and Pelvic Floor Diseases, The Sixth Affiliated Hospital, Sun Yat-sen University, Guangzhou, Guangdong, P. R. China; Department of General Surgery, The Sixth Affiliated Hospital, Sun Yat-sen University, Guangzhou, Guangdong, P. R. China; Department of Gastroenterology, The Sixth Affiliated Hospital, Sun Yat-sen University, Guangzhou, Guangdong, P. R. China; Guangdong Provincial Key Laboratory of Colorectal and Pelvic Floor Diseases, The Sixth Affiliated Hospital, Sun Yat-sen University, Guangzhou, Guangdong, P. R. China; Department of Gastroenterology, The Sixth Affiliated Hospital, Sun Yat-sen University, Guangzhou, Guangdong, P. R. China; Guangdong Provincial Key Laboratory of Colorectal and Pelvic Floor Diseases, The Sixth Affiliated Hospital, Sun Yat-sen University, Guangzhou, Guangdong, P. R. China; Department of Gastroenterology, The Sixth Affiliated Hospital, Sun Yat-sen University, Guangzhou, Guangdong, P. R. China; Guangdong Provincial Key Laboratory of Colorectal and Pelvic Floor Diseases, The Sixth Affiliated Hospital, Sun Yat-sen University, Guangzhou, Guangdong, P. R. China; Department of Gastroenterology, The Sixth Affiliated Hospital, Sun Yat-sen University, Guangzhou, Guangdong, P. R. China; Guangdong Provincial Key Laboratory of Colorectal and Pelvic Floor Diseases, The Sixth Affiliated Hospital, Sun Yat-sen University, Guangzhou, Guangdong, P. R. China; Department of Gastroenterology, The Sixth Affiliated Hospital, Sun Yat-sen University, Guangzhou, Guangdong, P. R. China; Guangdong Provincial Key Laboratory of Colorectal and Pelvic Floor Diseases, The Sixth Affiliated Hospital, Sun Yat-sen University, Guangzhou, Guangdong, P. R. China; Department of Gastroenterology, The Sixth Affiliated Hospital, Sun Yat-sen University, Guangzhou, Guangdong, P. R. China; Guangdong Provincial Key Laboratory of Colorectal and Pelvic Floor Diseases, The Sixth Affiliated Hospital, Sun Yat-sen University, Guangzhou, Guangdong, P. R. China

**Keywords:** Crohn's disease, small bowel, classification, progression

## Abstract

**Background:**

Small bowel involvement is related to poor prognosis in Crohn’s disease (CD), which may be a potential marker to stratify patients with a high risk of progression. This study aimed to establish a novel location classification system for CD and to develop a predictive model for disease progression.

**Methods:**

Consecutive patients with non-stricturing/non-penetrating CD were retrospectively included in the Sixth Affiliated Hospital, Sun Yat-sen University (Guangzhou, P. R. China) between January 2012 and January 2018. Patients were classified into two groups according to disease location: small bowel involvement group and isolated colon group. The primary outcome was disease progression to stricturing or penetrating phenotypes. Progression-free survival was estimated using Cox proportional hazards regression analysis and Kaplan–Meier method.

**Results:**

A total of 463 patients were analysed, with a median follow-up time of 55.3 months. Patients with small bowel involvement had a higher risk of disease progression than those with isolated colon disease (hazard ratio = 1.998, *P *=* *0.007), while no differences were found between Montreal location classification and disease progression. Median progression-free survival was higher in the isolated colon group than in the small bowel involvement group (84.5 vs 77.3 months, *P *=* *0.006). Four independent factors associated with disease progression were identified: small bowel involvement, duration of onset of >1 year, deep mucosal ulcer, and C-reactive protein levels of ≥10 mg/L (all *P *<* *0.05). The nomogram model based on these factors showed good performance in predicting disease progression, with a C-index of 0.746 (95% confidence interval, 0.707–0.785).

**Conclusions:**

Classifying CD based on small bowel involvement and isolated colon was superior to the Montreal location classification for predicting disease progression.

## Introduction

Crohn’s disease (CD) is a chronic gastrointestinal inflammatory disease that exhibits a progressive, destructive, and heterogeneous nature [1]. It can affect any part of the gastrointestinal tract from the oral cavity to the anus, with the most commonly affected areas being the terminal ileum and colon [2].

Studies have suggested that the location of the disease was associated with CD progression [3, 4]. Currently, the location classification in the Montreal system is widely used in adult CD [5]. Before the Montreal classification was established, small bowel imaging techniques were not well established. Namely, gastroscopy and colonoscopy were the most commonly used tools to identify disease location rather than the currently used tools such as computed tomography enterography (CTE) and magnetic resonance enterography (MRE) [5]. However, with the widespread adoption of small bowel imaging technology and endoscopy, more patients have been found to have small bowel involvement than previously thought. In an epidemiological study, approximately three-quarters of patients had small intestine lesions [6]. According to the current guideline, routine small bowel imaging is recommended for newly diagnosed CD patients [7]. It was also highlighted that location was important for disease characteristics and prognosis [8].

Recent studies have explored the clinical risk factors that predicted rapid progression and early surgery in CD, and developed models to predict prognosis [9–11]. These studies had shown that CD patients with small intestinal lesions had more severe disease and require more aggressive treatment than those with colonic CD. Involvement of the jejunum and extensive disease were the main factors predicting a poor prognosis in newly diagnosed CD [12, 13]. Additionally, patients with isolated small bowel CD were likely to have a long duration of onset to diagnosis, which may cause severe histological injury, worse clinical outcomes, and increased probability of surgery [14]. All these findings supported the need to verify an improved location classification system based on small bowel involvement.

In this study, we proposed a redesigned classification system that distinguished between small bowel involvement and isolated colonic CD, and developed a predictive model for disease progression based on this new classification.

## Methods

### Study population and design

This was a retrospective, single-center, observational cohort study. Patients newly diagnosed with CD between January 2012 and January 2018 were enrolled in the Sixth Affiliated Hospital, Sun Yat-sen University (Guangzhou, P. R. China). The diagnosis of CD was confirmed according to the current guidelines [15, 16], which is based on a combination of clinical, endoscopic, radiologic, and histological investigation. The inclusion criteria were as follows: (i) patients with non-stricturing/non-penetrating disease (B1) behavior; (ii) patients were followed up for ≥1 year; and (iii) patients with CTE/MRE and endoscopy evaluations at baseline and during follow-up. The exclusion criteria were as follows: (i) patients with malignancy; (ii) patients with a history of bowel resection; and (iii) patients with intestinal tuberculosis. Patients were categorized into two groups based on disease location: small bowel involvement and isolated colon involvement. The study protocol was approved by the Ethics Committee of the Sixth Affiliated Hospital, Sun Yat-sen University (approval No. 2021070).

### Definitions

Disease location and behavior were classified according to the Montreal classification [5]. Briefly, stricturing disease (B2) was defined by the combination of luminal narrowing, wall thickening, and pre-stenotic dilatation in CTE/MRE; penetrating disease (B3) was characterized by a fistulizing disease with abscesses or fistulas in an adjacent organ (not perianal fistula) [17, 18]. Small bowel involvement was defined as lesions in any part of the small intestine, including the duodenum, jejunum, ileum, or ileocecal valve. Isolated colon involvement was defined as the presence of lesions only in the colon, without any small intestine involvement. The Harvey–Bradshaw Index (HBI) measures clinical disease activity and an HBI score of >4 indicates active disease [19, 20].

### Outcome measures

The primary outcome of this study was disease progression to stricturing or penetrating phenotypes (B2 or B3) during follow-up. Disease progression was confirmed by endoscopy, radiology, or surgery. Patients who underwent bowel resection surgery without evidence of stricturing or penetrating disease were not considered as having disease progression. The secondary end point was the CD-related surgery.

### Data collection and follow-up

Demographic and clinical data were collected from medical records, including age, sex, body mass index, surgical history, smoking status, family history of inflammatory bowel disease (IBD), duration of onset (the time between the onset of symptoms to the time of diagnosis), disease location, disease behavior, laboratory tests, endoscopic findings, imaging results (the biggest bowel wall thickness), and treatment medications. In particular, C-reactive protein (CRP) of ≥10 mg/L is correlated with CD activity. HBIs were collected to reflect disease activity. Data on disease progression events and follow-up times were also recorded. Patients were followed up at IBD clinics or through telemedicine every 3 months. Follow-up CTE/MRE and endoscopy were conducted according to the treatment in the induction period. Usually, the first evaluation was conducted at 12–24 weeks after therapy. In the maintenance stage, the evaluation was conducted every 1–2 years.

### Statistical analysis

Statistical analysis was performed using SPSS software version 25.0 (IBM Corp., Armonk, NY, USA) and R software version 4.2.1 (R Foundation for Statistical Computing, Vienna, Austria). Continuous variables are expressed as mean ± standard deviation or median (interquartile range), and were compared using Student’s *t*-test or Mann–Whitney *U* test. Categorical variables are expressed as frequencies and percentages, and were compared using the chi-square or Fisher’s exact test. Survival analysis was performed using the Kaplan–Meier method and log-rank test. Univariate and multivariate Cox regression analyses were performed to identify the risk factors associated with disease progression. Variables with a *P-*value of <0.05 in univariate analysis were included in multivariate analysis. A nomogram model was constructed based on the significant risk factors using the rms package in R software. The performance of the nomogram model was evaluated by using the C-index, receiver-operating characteristic (ROC) curves, and calibration curves. A two-sided *P*-value of <0.05 was considered statistically significant.

## Results

### Patient characteristics

**Figure 1.**
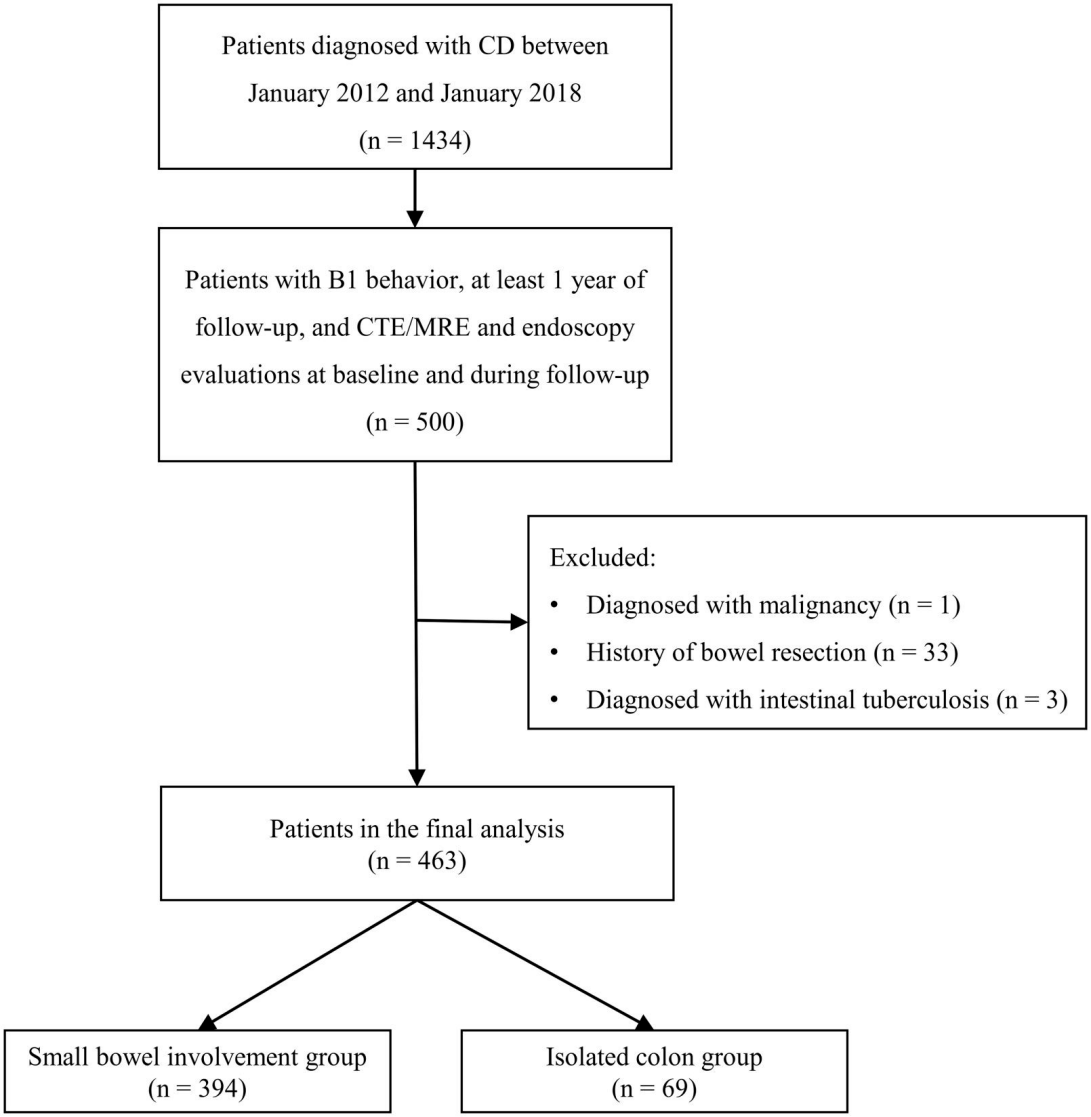
Flowchart of the study. CD = Crohn’s disease, B1 = non-stricturing/non-penetrating, B2 = stricturing disease, B3 = penetrating disease, CTE = computed tomography enterography, MRE = magnetic resonance enterography.

A total of 463 CD patients with B1 behavior were enrolled in this study (Figure 1). The median follow-up time was 55.3 (38.7–82.7) months. According to the Montreal classification, the most common disease location was L3/L3+L4 (51.2%), followed by L2/L2+L4 (23.1%), L1/L1+L4 (18.6%), and L4 (7.1%). Based on disease location, CD patients were classified into two groups: those with small bowel involvement (85.1%, *n *=* *394) and isolated colonic involvement (14.9%, *n *=* *69) (Table 1). The two groups exhibited comparable characteristics, including age, gender, and perianal disease. However, significant variations in inflammatory markers were observed between the two groups. Patients with isolated colonic CD had higher levels of inflammatory markers, including CRP (*P *=* *0.046) and erythrocyte sedimentation rates (*P *=* *0.002), than those with small bowel involvement CD. In contrast, patients with isolated colonic CD had lower albumin levels (*P *<* *0.001), hematocrit (*P *<* *0.001), and hemoglobin levels (*P *<* *0.001). However, there was no significant difference in the duration of onset between the small bowel involvement group and the isolated colonic group (*P *=* *0.494).

**Table 1.** Demographic and clinical features of small bowel involvement and isolated colon of Crohn’s disease

Characteristics	Small bowel involvement (*n *=* *394)	Isolated colon (*n *=* *69)	*P-*value
Male	293 (74.4)	46 (66.7)	0.183
Onset age			1.000
A1	83 (21.1)	14 (20.3)	
A2	291 (73.9)	51 (73.9)	
A3	20 (5.1)	4 (5.8)	
Glucocorticoids after diagnosis	123 (31.2)	28 (40.6)	0.126
Biologics after diagnosis	166 (42.1)	30 (43.5)	0.835
Immunomodulators after diagnosis	145 (36.8)	24 (34.8)	0.748
Mucosal ulcer			0.166
Deep	135 (34.3)	18 (26.1)	
Superficial	242 (61.4)	45 (65.2)	
No ulcer	17 (4.3)	6 (8.7)	
Duration of onset			0.494
≤1 year	188 (47.7)	36 (52.2)	
>1 year	206 (52.3)	33 (47.8)	
Perianal disease	277 (70.3)	51 (73.9)	0.543
Extra-intestinal manifestations	67 (17.0)	14 (20.3)	0.508
Need for intestinal surgery	70 (17.8)	5 (7.2)	0.029
HBI score at 1 year ≤ 4	180 (45.7)	46 (66.7)	0.001
BWT, mm, median (IQR)	9.0 (7.0–11.0)	9.0 (7.0–10.0)	0.894
WBC, × 10^9^/L, median (IQR)	7.57 (5.75–9.88)	7.95 (6.32–10.07)	0.290
Hematocrit, median (IQR)	0.37 (0.33–0.41)	0.35 (0.31–0.38)	<0.001
Hemoglobin, g/L, median (IQR)	120.0 (105.0–133.0)	108.0 (96.2–121.0)	<0.001
Albumin, g/L, median (IQR)	39.4 (35.3–43.1)	35.8 (30.4–40.4)	<0.001
BMI, Kg/m^2^, median (IQR)	18.3 (16.6–20.2)	18.0 (16.3–19.6)	0.202
ESR, mm/h, median (IQR)	34.0 (16.0–54.8)	51.0 (31.0–75.0)	0.002
CRP, mg/L, median (IQR)	16.0 (4.8–36.2)	20.9 (9.3–44.9)	0.046

A1, ≤16 years; A2, 17–40 years; A3, >40 years. BMI = body mass index, BWT = the biggest bowel wall thickness, CRP = C-reactive protein, ESR = erythrocyte sedimentation rate, HBI = Harvey–Bradshaw Index, IQR = interquartile range, WBC = white blood cell.

### Disease progression

**Figure 2.**
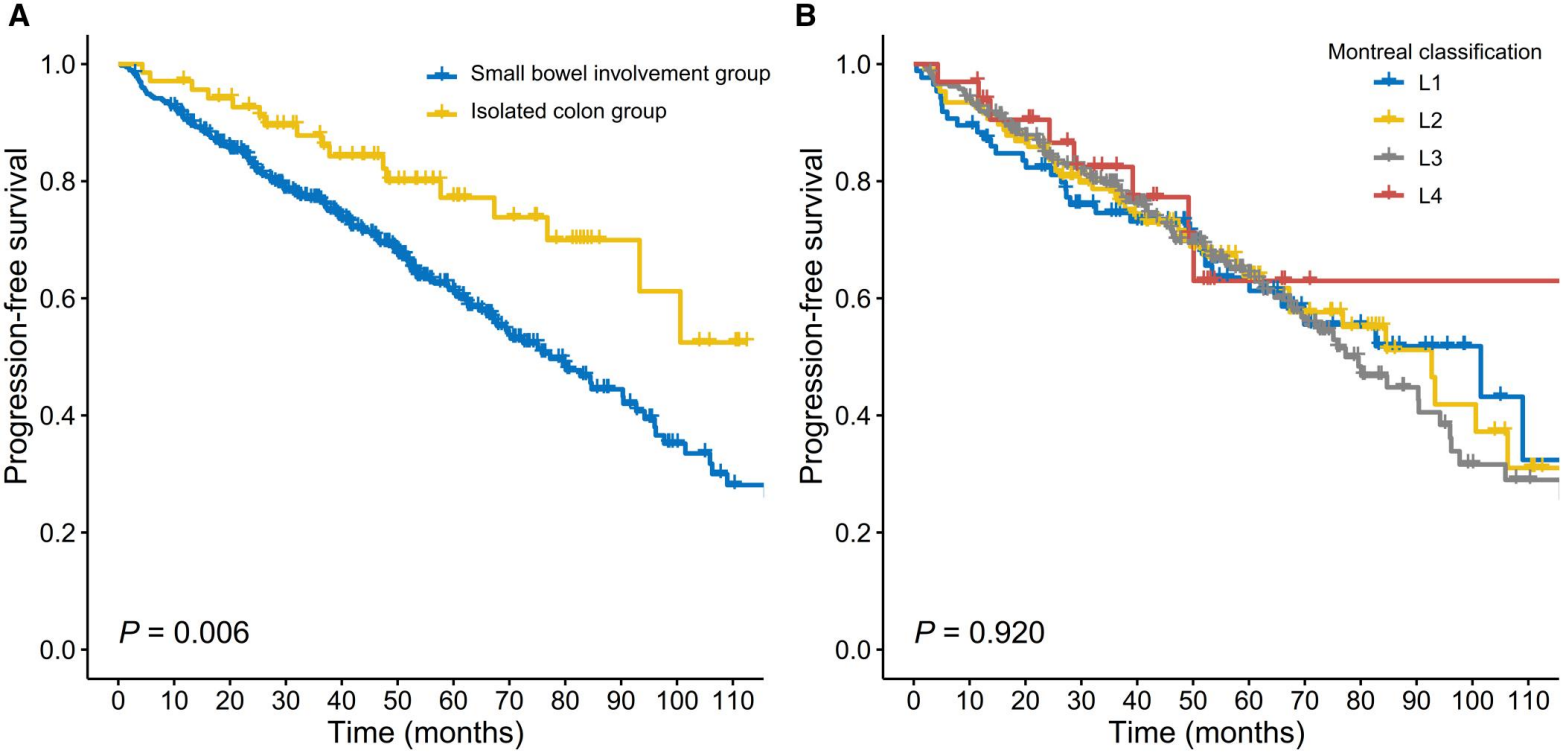
Kaplan–Meier curves for progression-free survival. (A) Kaplan–Meier curve for progression-free survival based on the novel location classification. (B) Kaplan–Meier curve for progress-free survival based on the Montreal classification.

Of 463 patients, 176 (38.0%) experienced progression during the follow-up. The cumulative incidences of progression at 1-, 2-, and 5-year follow-ups were 8.4% (39/463), 15.1% (70/463), and 29.4% (136/463), respectively. Kaplan–Meier curves showed that patients with small bowel involvement had a higher risk of disease progression than those with isolated colon involvement [hazard ratio (HR) = 1.998, *P *=* *0.007] (Table 2). The median progression-free survival was 77.3 [95% confidence interval (CI), 66.5–88.1] months in the small bowel involvement group and 84.5 (95% CI, 73.5–95.5) months in the isolated colon group (Figure 2A). Of note, in the Montreal classification, the Kaplan–Meier curves did not demonstrate differences between disease locations and CD progression (*P *=* *0.920) (Figure 2B).

**Table 2.** Factors associated with the possibility of progression in patients with Crohn’s disease

Characteristics	Univariate analysis		Multivariate analysis	
	HR (95% CI)	*P*	HR (95% CI)	*P*
Gender (male vs female)	1.121 (0.812–1.548)	0.487		
Onset age (years)				
≤16	Reference			
17–40	1.271 (0.897–1.801)	0.178		
>40	1.124 (0.565–2.239)	0.739		
BMI (kg/m^2^)				
≥18.5	Reference		Reference	
<18.5	1.824 (1.333–2.495)	<0.001	1.017 (0.704–1.469)	0.928
Montreal classification				
L2/L2+L4	Reference			
L1/L1+L4	1.024 (0.651–1.611)	0.918		
L3/L3+L4	1.065 (0.739–1.535)	0.736		
L4	0.836 (0.391–1.786)	0.643		
Location				
Isolated colon	Reference		Reference	
Small bowel involvement	1.998 (1.210–3.298)	0.007	1.767 (1.022–3.055)	0.041
Duration of onset >1 year	2.073 (1.515–2.837)	<0.001	1.560 (1.084–2.245)	0.017
Deep mucosal ulcer	6.561 (4.612–9.334)	<0.001	4.934 (3.286–7.408)	<0.001
BWT (1 mm increased)	1.110 (1.063–1.160)	<0.001	1.025 (0.971–1.082)	0.366
Glucocorticoid after diagnosis	1.031 (0.755–1.407)	0.847		
Extra-intestinal manifestations	1.050 (0.715–1.540)	0.804		
Perianal surgery	1.067 (0.791–1.441)	0.670		
CRP ≥ 10 mg/L	2.209 (1.553–3.142)	<0.001	1.810 (1.213–2.701)	0.004
Perianal lesions	1.046 (0.762–1.437)	0.780		

BMI = body mass index, BWT = the biggest bowel wall thickness, CI = confidence interval, CRP = C-reactive protein, HR = hazard ratio, L1 = terminal ileum, L2 = colon, L3 = ileocolon, L4 = upper gastrointestinal tract.

The data on therapies including steroids, immunosuppressants, and biologics in the follow-up period are shown in Table 1. They showed no significant differences between the two groups. However, during the first year of follow-up, more patients in the isolated colon group achieved clinical remission than those in the small bowel involvement group (66.7% vs 45.7%, *P *=* *0.001). In addition, patients with small bowel involvement showed a higher rate of CD-related surgery than those with isolated colon disease during follow-up (17.8% vs 7.2%, *P *=* *0.029).

In the multivariate analysis, stricturing and penetrating complications were significantly associated with small bowel involvement, the duration of onset, deep mucosal ulcers, and CRP of ≥10 mg/L at diagnosis (Table 2).

### Development, evaluation, and validation of the nomogram model

**Figure 3.**
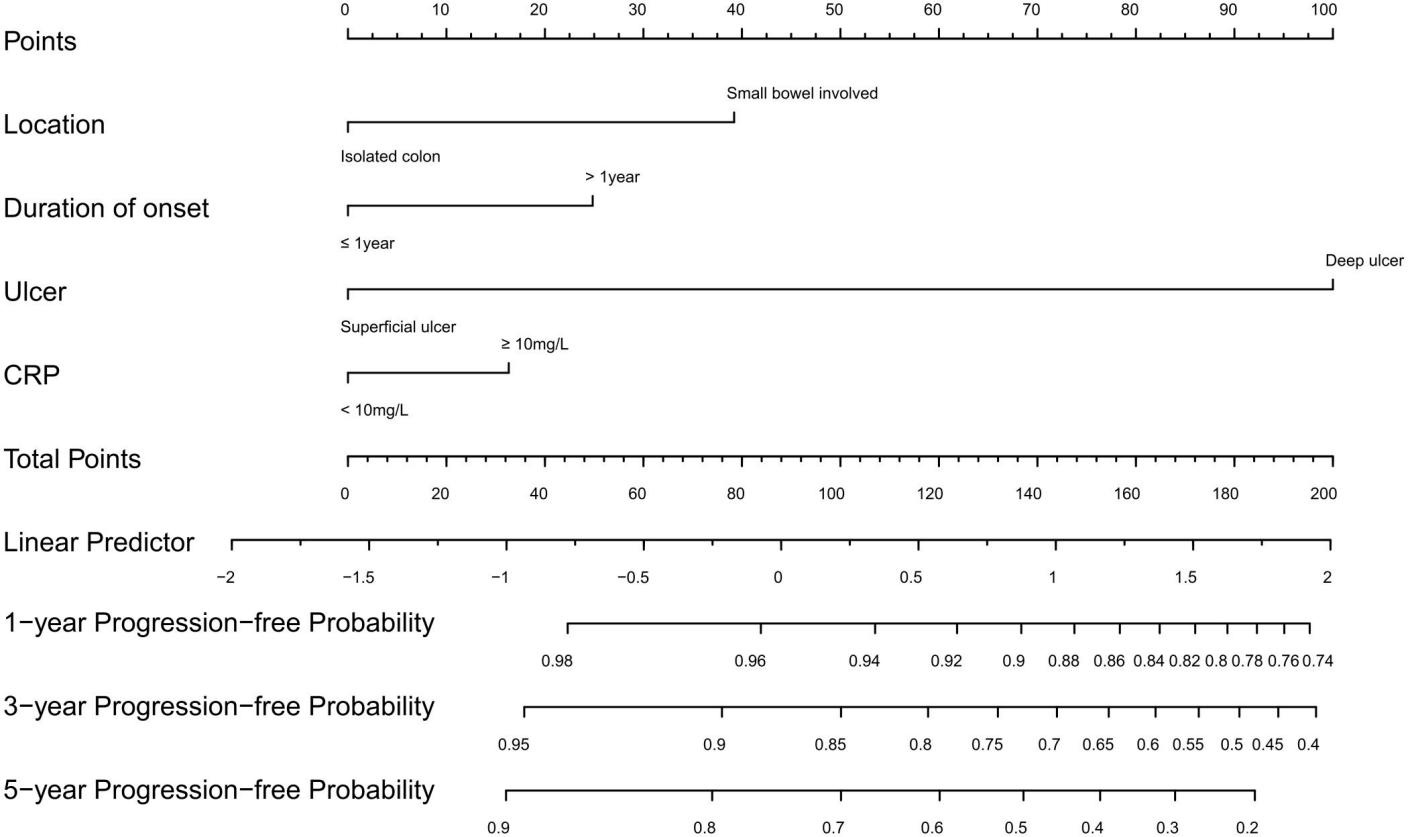
Nomogram model for predicting progression-free survival based on the novel location classification in non-stricturing/non-penetrating Crohn's disease

To further verify the advantages of the new classification, a nomogram model was established to predict complicated disease behaviors (Figure 3). The nomogram allows clinicians to easily obtain the probability of disease progression by calculating the total number of points and matching them vertically downward to the risk of progression at 1-, 3-, and 5-year follow-ups.

**Figure 4.**
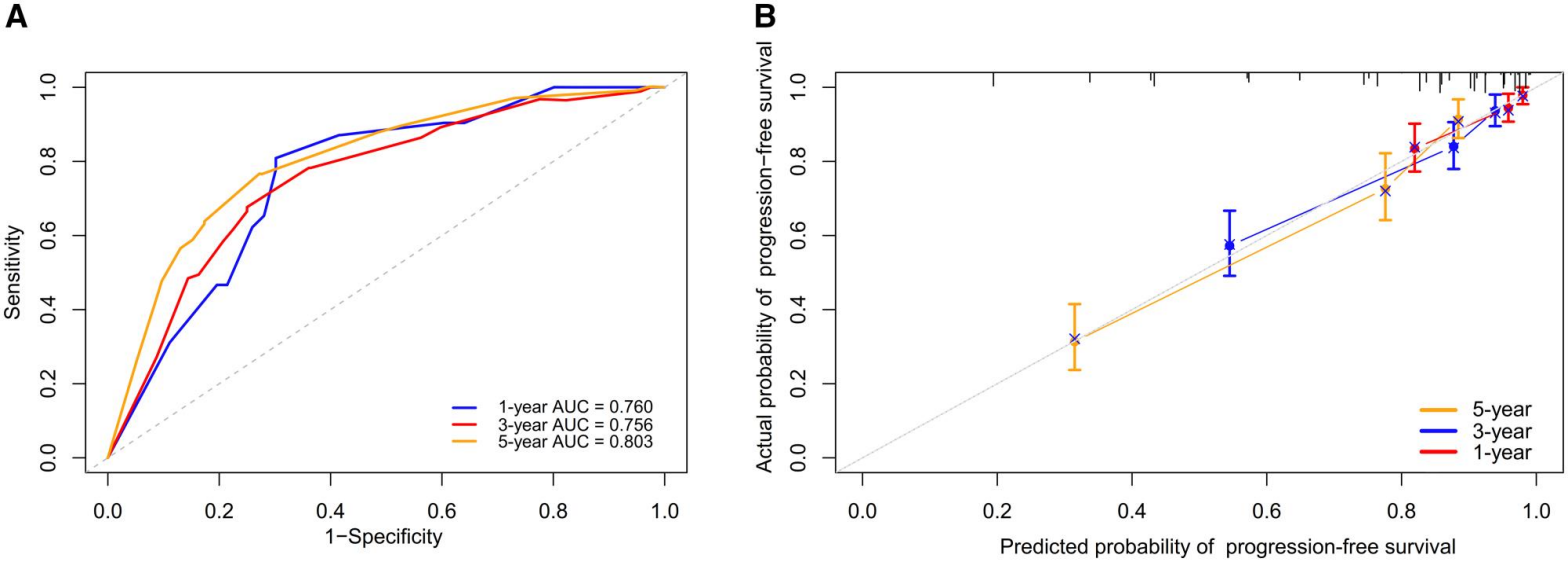
ROC curves and calibration curves of the predictive model. (A) ROC curves show the discriminatory ability of the nomogram for predicting the 1-, 3-, and 5-year progression-free survival in Crohn's disease patients. (B) Calibration curves to validate the nomogram for 1-, 3-, and 5-year progression-free of Crohn's disease patients. AUC = area under the curve, ROC = receiver-operating characteristic.

To evaluate the performance of the predictive model, we used ROC curves. The area under the curves for progression-free survival at 1, 2, and 5 years were 0.760, 0.756, and 0.803, respectively (Figure 4A). The calibration curve showed that the model agreed well with the calculated risk and the observed outcomes (Figure 4B). Furthermore, the C-index of the nomogram is 0.746 (95% CI, 0.707–0.785). These results indicated that the predictive model had good performance and applicability.

## Discussion

In the present study, we found that the novel location classification system based on small bowel involvement could better predict disease progression and was superior to the traditional location classification in the Montreal system. We also developed a nomogram model based on the new location classification system and other risk factors to assess the risk of progression to severe phenotypes in CD patients. These findings have important clinical implications for personalized treatment and management of CD.

Our study highlighted the importance of accurately classifying CD location and found that small bowel involvement was a key predictor of disease prognosis. This is consistent with the previous studies showing that small bowel involvement was associated with a more severe disease course, higher rates of complications, and increased need for surgery [14, 17, 21, 22]. The possible explanation for this was that the small intestine plays a crucial role in the pathogenesis of CD, as it is the site of antigen presentation, immune activation, and bacterial translocation [18, 23]. Moreover, small bowel lesions are often difficult to detect and treat, leading to delayed diagnosis and inadequate therapy [8, 24]. It was worth noting that CD patients with small bowel involvement exhibited milder clinical manifestations at baseline, with lower CRP and erythrocyte sedimentation rates, and higher albumin and hemoglobin levels. However, the isolated colon group achieved a higher rate of clinical remission (66.7% vs 45.7%, *P *=* *0.001) and a lower rate of CD-related surgery than the small bowel involvement group during follow-up. These findings also indicated that, compared with isolated colonic CD, CD with small intestinal lesions may exhibited milder clinical symptoms but earlier behavioral progression. Therefore, identifying patients with small bowel involvement early may help optimize treatment strategies and prevent disease progression.

We also found that duration of onset of >1 year, deep mucosal ulcers, and elevated inflammatory marker levels were independent risk factors for disease progression. These factors reflect the chronic nature, severity, and activity of CD, respectively. Previous studies have also reported similar results [9–11, 25]. These factors may be used as indicators for initiating more aggressive treatment or closer monitoring in CD patients.

Based on the new location classification system and other risk factors, we constructed a nomogram model that could effectively and accurately predict disease progression in CD patients. The nomogram model was a graphical tool that could estimate individualized risk by integrating multiple variables [26]. Compared with conventional regression models, nomogram models are more user-friendly and intuitive, as they can directly translate the values of each variable into a total score and a corresponding probability. Nomogram models have been widely used in various fields of medicine, especially in oncology [27, 28]. However, few studies have applied nomogram models to predict prognosis in CD [29]. Similarly, in the CDPATH risk assessment tool, the small bowel involvement was identified as a risk factor for CD-related complications, which was consistent with our study [30]. However, our study included a larger sample size for verification, and the nomogram model showed good performance and accuracy. The ROC curves and calibration curves also demonstrated good discrimination and calibration abilities of the nomogram model. Therefore, our nomogram model may be a useful tool for clinicians to assess the risk of disease progression in CD patients, and to guide individualized treatment decisions.

Our study has several strengths. First, we proposed a novel location classification system for CD based on small bowel involvement, which has not been reported before. Second, we developed a nomogram model based on the new location classification system and other risk factors to predict disease progression in CD patients. However, our study also has some limitations. First, this was a retrospective study. Therefore, selection bias and confounding factors may exist. Some factors such as the exact number of endoscopic ulcers, changes of treatment, and disease remission rates during follow-up were difficult to be accurately reviewed. Second, we did not include some potential risk factors for disease progression, such as genetic factors, microbiota composition, environmental factors, or medication adherence. Third, we did not compare our nomogram model with other existing models or scoring systems for predicting prognosis in CD. Further studies are needed to confirm and generalize our findings.

## Conclusion

In conclusion, we established a novel location classification system for CD based on small bowel involvement and further developed a nomogram model to predict disease progression based on the new classification. The findings suggested that classifying CD based on small bowel involvement and isolated colonic disease may be superior to the traditional location classification in the Montreal system for predicting disease progression. The nomogram model may be a useful tool for clinicians to assess the risk of disease progression in CD patients, and to guide individualized treatment decisions at an early stage. However, further studies are needed to validate our results in larger and more diverse populations.

## Authors’ Contributions

H.G., J.T., K.C., and X.G. contributed to the conception and design of the study. H.G., X.Q., M.Lin, M.Li, Q.Y., and Z.H. contributed to data acquisition. H.G., J.T., and X.Q. performed the statistical analysis. H.G. drafted the manuscript. All authors read and approved the final version of the manuscript.
